# Mindfulness and MBCT-vision (mindfulness-based cognitive therapy modified for visual symptoms) for visual snow syndrome: a therapeutic perspective

**DOI:** 10.3389/fneur.2025.1596642

**Published:** 2025-07-01

**Authors:** Sui H. Wong, Janet Wingrove

**Affiliations:** ^1^Kings College London, London, United Kingdom; ^2^Guys and St Thomas’ NHS Foundation Trust, London, United Kingdom; ^3^Moorfields Eye Hospital NHS Foundation Trust, London, United Kingdom; ^4^Institute of Neurology, University College London, London, United Kingdom; ^5^South London and Maudsley NHS Foundation Trust, London, United Kingdom

**Keywords:** visual snow syndrome, mindfulness, attention network, salience network, default mode network, lifestyle intervention, mindfulness-based intervention

## Abstract

Visual snow syndrome (VSS) is a neurological disorder characterized by intrusive visual symptoms and associated with dysregulation in brain networks, including the Salience Network, Default Mode Network, and thalamocortical circuits. This perspective paper examines the application of mindfulness-based cognitive therapy modified for visual symptoms (MBCT-vision) as an intervention for VSS. Drawing on clinical experience, our recent open-label study, and ongoing randomized controlled trial, we propose that VSS symptoms may perpetuate through attentional mechanisms, including heightened vigilance and threat attribution. We outline how mindfulness practices in MBCT-vision address these processes by enhancing attentional flexibility, increasing metacognitive awareness, and fostering a non-reactive stance toward symptoms. The group-based format of MBCT-vision provides additional therapeutic benefits through shared understanding and validation. We discuss potential neuroplastic mechanisms underlying observed improvements, particularly involving the Default Mode Network. This paper advances the understanding of mindfulness mechanisms in VSS and provides a foundation for developing comprehensive, evidence-based approaches that integrate neurobiological insights with person-centered therapeutic strategies for this challenging condition.

## Introduction

Visual snow syndrome (VSS) is a disorder involving brain network dysregulation, in the presence of normal ophthalmological and neurological examinations (1–3). VSS causes intrusive and disruptive visual symptoms, including “visual snow”: so-called because of the resemblance to “TV show” (analog TV static due to background electromagnetic radiation). Also reported to be intrusive are: after-images, trailing of moving images and entoptic phenomena (arising within the eye itself) such as flashes and floaters, and the blue field entoptic phenomenon (caused by the movement of white blood cells through retinal vessels). Beyond visual disruption, individuals often experience significant non-visual symptoms, including depersonalization and tinnitus (4).

Our recent open-label study using a mindfulness-based intervention, mindfulness-based cognitive therapy modified for visual symptoms (MBCT-vision), improved self-reported VSS symptoms with associated changes on functional magnetic resonance imaging (fMRI) (5).

Building on these findings, we share our perspective on how mindfulness can benefit people with VSS. Our perspective is informed by clinical experience treating individuals with VSS, including observations from our open-label study (5) and ongoing randomized controlled trial (ClinicalTrials.gov: NCT06018103).

**Figure 1:**
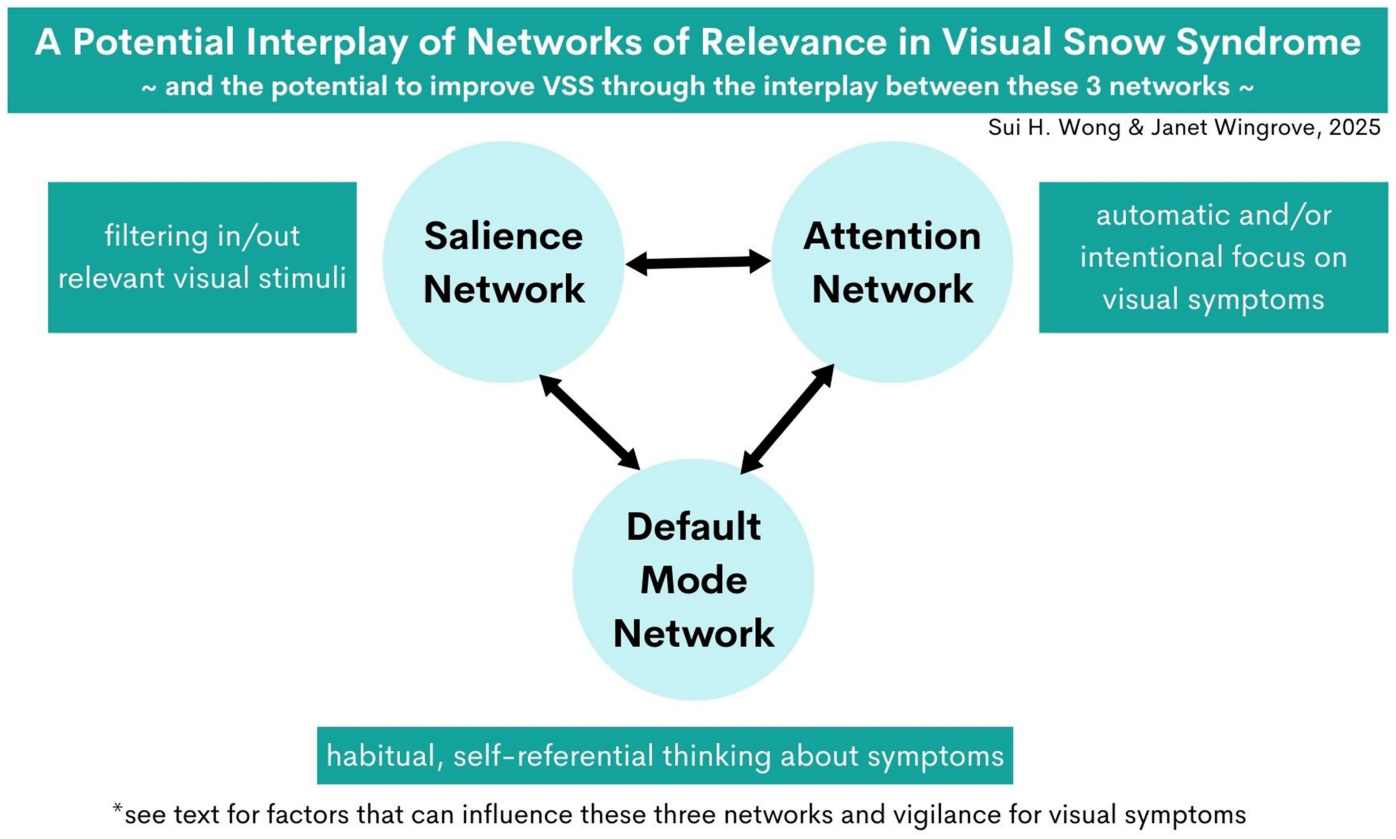
A potential interplay of networks of relevance in Visual Snow Syndrome (VSS), and the potential to improve VSS through the interplay between these three networks.

This paper aims to advance the understanding of mindfulness mechanisms in VSS and provide a foundation for developing comprehensive, evidence-based treatment approaches. By examining the intersection of salience and attentional networks, mindfulness practices, and VSS symptomatology, we offer insights into a holistic, person-centered therapeutic strategy for this challenging condition (summarized in Box 1 and Figure 1).

BOX 1 **Holistic model of therapeutic approaches to support people with VSS**

**Table 1:** 

Concepts*	Potential areas for therapeutic approaches, personalized to support people with VSS
Vision is a key part of the vigilance & attention systems, for human safety & wellbeing. The visual phenomena experienced in VSS can be transiently noticed as a normal phenomenon and filtered out (by the salience/attentional networks) as insignificant in non-VSS sufferers.	
In VSS, salience/attentional networks are affected, making it difficult to filter out visual symptoms	Supporting the salience network, with: The treatment of underlying contributing causes, if present, e.g., migraine; sleep disruption; concussion; stress; mental health conditions (e.g., anxiety, attention deficit hyperactivity disorder, post-traumatic stress disorder); viral infections; pain. Lifestyle modifications to improve brain health and wellbeing, e.g., sleep, caffeine, alcohol, physical activity, nutrition, metabolic health, stress management, mindfulness-based practices.
In some people with VSS, the attentional bias to visual symptoms may be exacerbated by attempts to investigate and monitor them	Supporting this: early diagnosis & understanding of VSS treatment of underlying health anxiety that may be driving the initial conscious monitoring of visual symptoms
This in turn affects the “filtering out” of visual symptoms.	Mindfulness-based approaches can create flexibility in changing the attentional processes, from automatic to more conscious control
The locking onto visual symptoms may be driven by vigilance for danger.	Support this through the changing of relationship to VSS symptoms, changing it from one of aversion or reactivity, to a more neutral response, to allow the “letting go” of visual symptom as unimportant. Examples of holistic approaches to support this: Metacognitive awareness of thought patterns contributing to this, through therapeutic approaches such as Mindfulness Based Cognitive Therapy Wellbeing practices, e.g., the above-mentioned lifestyle modifications, and mindfulness-based approaches Group support including peer-to-peer and facilitated discussions One-to-one therapeutic support

*These approaches can support neuroplasticity and modulating the heightened visual networks in VSS. NB: see full text for discussions and references.

## Attentional processes in VSS and the role of mindfulness

VSS is a brain network disorder (2). Current evidence implicates three key networks: the salience network (SN), default mode network (DMN), and thalamocortical circuits (5–7). These networks play crucial roles in attention regulation, vigilance maintenance, and the processing of emotional and behavioral responses (8–11).

Considering VSS through the lens of attention theory provides valuable insights into symptom perpetuation and potential therapeutic approaches. An established model of the attentional system considers three main components: alerting; orienting; and executive control (12, 13). Alerting refers to achieving and maintaining a state of heightened sensitivity to incoming stimuli. Orienting involves selecting specific information from sensory input. Executive control is responsible for monitoring and resolving conflicts among thoughts, feelings, and responses.

Attention is a selective process that allows us to focus on specific stimuli while filtering out irrelevant information (13, 14). This can be influenced by factors including self-relevance (15) and stimulus selection for the promotion of survival and wellbeing (14).

The selection process operates through a combination of automatic and controlled mechanisms (12, 13). Automatic processes occur rapidly and without conscious effort, while controlled processes are deliberate and require conscious awareness. However, newer research suggests that these two processes can become more flexible and context-dependent, contrary to classical theories that portrayed them as a rigid distinction (16).

This potential for flexibility can help us understand the experience of VSS in some people, e.g., symptom monitoring which become more automatic over time; and the potential for therapeutic advances, e.g., changing automatic processes to become more deliberate with mindfulness practices, thereby an opportunity for change and neuroplasticity to improve VSS.

VSS symptoms may perpetuate through an interplay between the salience and attentional networks, with influence by the thalamocortical circuits. Disruptions to these lead to an increased awareness of visual symptoms that would normally be “filtered out.” The visual phenomenon experienced in VSS can be transiently noticed as a normal phenomenon and “filtered out” as insignificant in non-VSS sufferers. However, this “filtering out” process can be affected by various factors such as migraines, sleep deprivation, anxiety or stress (17–20).

The pathophysiological cascade in VSS may begin with an increased awareness of a range of visual phenomena, leading to attentional orientation toward these phenomena. This initial awareness of visual symptoms then leads on to an orienting of attention to visual symptoms, which can become more automatic, if unmanaged.

This process can become self-perpetuating through two mechanisms:

- threat attribution: interpreting the symptoms as a source of threat because of the way they interfere with current tasks, or because of the perception that they signify potential dangers such as blindness;
- monitoring behavior: e.g., to check if the symptoms are getting worse or better; to describe them to healthcare practitioners; or to get a diagnosis.

The presence of co-morbid health anxiety may further perpetuate this orienting of attention to visual symptoms. The concept that VSS is perpetuated by maladaptive attentional processes is not yet well established in the literature and is based on our perspective with the rationale outlined above. This concept has been shaped by our discussions with people about the onset of their VSS symptoms, their initial reactions, and subsequent monitoring habits. We are currently undertaking a qualitative study to further understand this and will be publishing the results in due course.

## Heightened vigilance and attention

This heighted awareness of VSS symptoms may be linked with a heightened vigilance to threat. This heightened vigilance and may explain the broader phenomenology of VSS beyond visual disturbances, such as anxiety, feeling of disconnection from oneself or reality, and cognitive difficulties (4).

Additionally, aversion to VSS symptoms may lead to deliberate efforts to ignore or suppress them, which may further impair attentional control (17, 21). This further perpetuates a cycle of heightened vigilance: visual symptoms worsen anxiety, mood and sleep, which in turn affects the salience network function, further impairing perceptual filtering and leading to increased symptom awareness.

Research in cognitive psychology has shown that attention allocation is significantly influenced by personal relevance and perceived threat (22). This is relevant in VSS, as the persistent and intrusive nature of VSS symptoms carry high personal relevance and potential threat value, further creating challenges for attention regulation.

Traditional advice to “ignore” symptoms contradicts basic attention mechanisms, as the skills required such as disengaging from visual stimuli and focus-switching (12, 13) become especially challenging when symptoms are persistent and perceived as threatening.

This is where mindfulness-based approaches can play a crucial role in the treatment of VSS, by directly addressing these attentional challenges. Rather than attempting to suppress symptom awareness, mindfulness practices help break the cycle of heightened vigilance and symptom perpetuation in VSS by modifying fundamental attention processes. This approach enhances attention regulation capabilities while developing non-reactive awareness.

## Mindfulness and MBCT-vision for VSS

Mindfulness is defined as “the awareness that arises from paying attention in a particular way; on purpose, in the present moment and non-judgmentally.” (23). Mindfulness practices often incorporate the qualities of non-judgment, kindness, curiosity, friendliness and openness (24, 25).

MBCT-vision is a mindfulness-based intervention delivered as a structured group-learning program over 8 weeks. MBCT-vision is a modification of the Mindfulness-Based Cognitive Therapy (MBCT) program created by Segal, Williams, Teasdale (26), built on the Mindfulness-Based Stress Reduction (MBSR) program created by Kabat-Zinn (23).

In the MBCT program, participants learn a repertoire of mindfulness practices, structured around weekly themes with practices that include sitting, movement and informal practices during daily activities. Each theme builds on the next over 8 weeks. In addition to mindfulness skills, participants learn meta-cognitive skills, e.g., awareness of thought patterns that may be affecting their wellbeing.

The groups meet once weekly for 8 weeks and are given daily home practices in between group meetings. The group meetings include guided mindfulness practices and structured discussions about their daily home practices, facilitating experiential and reflective learning.

In developing a mindfulness-based intervention for VSS, we chose MBCT as the base structure, due to extensive research on MBCT as a clinical intervention. MBCT has been studied in numerous clinical trials and is recommended by the National Institute of Clinical Excellent in the United Kingdom for preventing recurrent depression (27). Additionally, MBCT has been shown to be effective in health anxiety (28), which in our experience may also be co-existent in VSS.

We modified MBCT into MBCT-vision for the treatment of VSS, by removing structured discussions about preventing depression. Instead, we included discussions about VSS; mindfulness and other lifestyle modifications for improving resilience and wellbeing (detailed further below); and guidance on modifications to mindfulness practices due to VSS symptoms where necessary.

Practices are taught as described in the original MBCT program, which includes body scan, focus on breath, mindful movement (similar to certain practices of Tai Chi or Yoga), and a technique called “3-step breathing space” which is a three step process of being aware of body sensations, emotions, thoughts, situation; followed by anchoring on the breath; and expanding awareness and attention to the wider present moment again. We also added additional sensory tactile practices such as tapping of the body to help with anchoring to the present moment, alongside the wider principles of breath and body, which we find particularly useful in the context of depersonalization symptoms.

## Mechanisms of mindfulness in VSS

The mindfulness practices taught in MBCT-vision help participant become better at noticing the connections between their body sensations, emotions, and thought processes.

Mindfulness practices can create a shift in how individuals relate to their symptoms by working with the attentional and emotional salience of VSS experiences. Mindfulness-based interventions have been shown to enhance attentional control and emotional regulation (29), allowing individuals with VSS to relate to their symptoms in a less reactive and more accepting manner.

By cultivating non-judgmental awareness and the ability to observe symptoms without automatically engaging with them, mindfulness may help reduce the distress associated with VSS and improve overall quality of life (23). This approach acknowledges the difficulty of “ignoring” symptoms while providing practical tools for managing attention and emotional responses to VSS experiences.

Mindfulness skills allow people with Visual Snow Syndrome (VSS) to:

- Practice disengaging attention from one stimulus and redirecting it to another, enhancing attentional flexibility (25).
- Increase awareness of attentional processes, including recognizing patterns of conscious, deliberate monitoring of symptoms and learning to disengage from this habitual focus (24).
- Cultivate the ability to bring other aspects of experience, particularly body sensations and other senses, into the foreground of awareness. This practice can create conditions for VSS symptoms to recede into the background of perception (30).
- Recognize habitual patterns of aversion toward symptoms and understand their consequences. This awareness allows individuals to develop a more balanced relationship with their VSS experiences (31).
- Learn to allow VSS symptoms to be present without excessive attachment (treating them as overly important) or aversion (responding with fear or tension). This approach fosters a more accepting and less reactive stance toward symptoms (23).

These mindfulness skills collectively contribute to a more adaptive way of relating to VSS symptoms, potentially reducing their impact on daily life and well-being.

## Group learning in MBCT-vision

The cognitive skills that participants develop through reflective learning during group discussions, help them identify patterns that may be perpetuating their symptoms. These discussions, when anchored in the experience of mindfulness practices, are integral to the process of changing their relationship to VSS.

Participants learn to observe their thoughts, emotions, and bodily sensations related to VSS with increased awareness and objectivity. This metacognitive perspective allows them to recognize automatic patterns of reactivity and develop more adaptive responses to their symptoms.

Moreover, the group setting provides a unique therapeutic environment. Feeling understood by the facilitators and sharing experiences with other participants who face similar challenges can significantly contribute to reducing the sense of threat associated with VSS symptoms. This shared understanding and validation can help normalize the experience of VSS and reduce the anxiety and isolation often associated with the condition.

The combination of mindfulness practices, cognitive skills, and group support creates a comprehensive approach to managing VSS symptoms. This multifaceted intervention aims to not only alleviate symptoms but also improve overall quality of life by fostering a more accepting and less reactive relationship with VSS experiences.

## Neuroplasticity and the default mode network with MBCT-vision in VSS

The approach of anchoring in mindful mind–body practices helps participants develop a sense of psychological safety, as part of the process of settling and reducing heightened vigilance.

The default mode network (DMN) is part of the vigilance system (32) and can be modified with mindfulness practices (33). This could be the process underlying the changes on fMRI following MBCT-vision in our recent study (5). We speculate that this could also link in with a recent neuroimaging study showing reduced serotonergic and glutaminergic connectivity in VSS (34), as both serotonin and glutamine neurotransmitters are involved in DMN (35).

A regular practice is recommended to promote long lasting changes in neural networks (29), which is what we encourage participants to do after the MBCT-vision program. Our feasibility study showed that self-reported outcome using a 0–10 visual analog scale was more significant at 3 months compared to 1 week following the MBCT-vision program (5). However, the limitation was that it was an open-label study. We are therefore currently delivering an RCT.

Our current RCT includes a secondary outcome measure at 1-year. We notice that with regular practice, participants become more skilled at reducing reactivity to their visual symptoms, have more agency and skill to shift their attention as needed.

## Toward a holistic, person-centered approach

Our treatment approach in MBCT-vision includes discussions on lifestyle measures that improves attentional control and executive function, i.e., improving the above-mentioned concept of the “filter.” Lifestyle modifications discussed include sleep hygiene, physical activity, nutrition, limiting excessive caffeine or alcohol, and stress management. These adjunctive topics covered in group discussions, while mindfulness remains the main focus of MBCT-vision.

The complex interplay between VSS and comorbid conditions necessitates a comprehensive treatment approach. Disruptions to the Salience Network, Thalamocortical Circuits, and Default Mode Network may be exacerbated by conditions such as migraine, anxiety, and sleep disorders (17–20). Therefore, effective management of these comorbidities becomes integral to VSS treatment success.

We advocate a holistic and personalized approach in the support of people with VSS. Our clinical experience suggests that some individuals benefit from personalized therapeutic support before engaging in group-based interventions. Individual therapy, particularly incorporating somatic or mind–body practices, can prepare participants for optimal engagement with the MBCT-vision program. This staged approach acknowledges the diverse needs of individuals with VSS and allows for appropriate therapeutic tailoring.

By simultaneously addressing network dysfunction, attention regulation, and contributing factors, this comprehensive framework aims to modify both symptoms and their underlying mechanisms, potentially leading to sustained improvement in clinical outcomes.

## Future directions

The emerging understanding of VSS as a network disorder, combined with preliminary evidence for mindfulness-based interventions, opens several promising avenues for future research.

Our ongoing randomized controlled trial, which includes a qualitative sub-study with structured interviews, will provide valuable insights into both the efficacy of MBCT-vision and the lived experience of participants. The upcoming publication of these findings will help refine our theoretical framework and treatment approach.

Critical areas for future investigation include the optimization of mindfulness-based interventions for VSS, lifestyle-based interventions, identification of predictive factors for treatment response, and exploration of potential synergies between MBCT-vision and other therapeutic modalities. Furthermore, research into interventions of shorter duration, which include a focus on the fears and experiences of living with VSS could be helpful.

Additionally, research examining the neurobiological and attentional mechanisms underlying mindfulness effects in VSS, particularly through advanced neuroimaging techniques and attention tasks, may reveal new therapeutic targets and guide treatment refinement.

We suggest that studies showing changes in visual networks in VSS (2, 34, 36) are likely a downstream effect of the salience, attention and default mode networks for the above-mentioned reasons. Therefore studies focusing on the latter networks will be of particular relevance.

The development of effective treatments for VSS requires a continued commitment to rigorous research methodology while maintaining sensitivity to individual patient needs. As our understanding of VSS pathophysiology and the role of attention networks continues to evolve, integration of these insights with clinical observations will be crucial for advancing therapeutic approaches. Through this combined basic science and clinical research agenda, we aim to enhance our ability to provide effective, personalized care for individuals living with VSS. We continue in our endeavors with our current RCT, due to complete in 2027; and future related work.

## Data Availability

The original contributions presented in the study are included in the article/supplementary material, further inquiries can be directed to the corresponding author.
